# Gender Differences in a Cohort of Children with Eating Disorders: A Retrospective Study

**DOI:** 10.3390/children12050652

**Published:** 2025-05-19

**Authors:** Isabella Tarissi de Jacobis, Elena Inzaghi, Elena Bozzola, Valeria Zanna, Cristina Mascolo, Sara Caterina Kupiec, Maria Rosaria Marchili

**Affiliations:** 1 Pediatric Unit, Bambino Gesù Children’s Hospital, IRCCS, 00165 Rome, Italy; isabella.tarissi@opbg.net (I.T.d.J.); cristina.mascolo@opbg.net (C.M.); mrosaria.marchili@opbg.net (M.R.M.); 2 Endocrinology and Diabetology Unit, Bambino Gesù Children’s Hospital, IRCCS, 00165 Rome, Italy; elena.inzaghi@opbg.net; 3 Child and Adolescent Neuropsychiatry Unit, Bambino Gesù Children’s Hospital, IRCCS, 00165 Rome, Italy; valeria.zanna@opbg.net; 4 Department of Pediatrics, University of Rome Tor Vergata, 00100 Rome, Italy; sarac.kupiec05@yahoo.com

**Keywords:** gender, anorexia nervosa, clinical, biochemical parameters, children

## Abstract

**Introduction**: Eating disorders (EDs) are severe psychiatric disorders that significantly impact the psychological and physiological well-being of affected individuals. Despite increasing prevalence in males over the past few decades, EDs are mainly considered a female disease. This retrospective study aims to evaluate the influence of gender on the clinical, laboratory, and developmental characteristics of EDs in pediatric patients. **Material and methods**: A retrospective study was conducted on patients referred to the EDs between 2019 and 2024 at Bambino Gesù Children’s Hospital, Rome, Italy. Gender differences were evaluated in the whole cohort and in a sub-cohort of males and females well matched for age. **Results**: In the whole cohort of 501 patients (age range 8–17 years), 45 were males and 456 were females. In male patients, EDs occurred at a younger age (12.65 vs. 14.65 years, *p* < 0.05). When considering the matched cohort, male patients showed more severe clinical conditions, as evidenced by a tendency to a lower BMI SDS at the onset, higher inflammatory parameters (ferritin and transaminase levels), and poorer nutritional status (vitamin D levels: 23.15 vs. 26.9 ng/mL, *p* < 0.05). However, male patients had a shorter hospital stay (14.8 vs. 19.9 days, *p* < 0.05) and showed a tendency to a greater clinical improvement. Differences in therapy were also observed, with fewer males receiving pharmacological treatment or nasogastric feeding. **Conclusions**: The results of the current study confirm the higher prevalence of EDs in females, even though they suggest EDs are not exclusively a female disease. Male gender may represent a risk factor for a worse clinical course and a younger onset. Further studies with a longer follow-up are required to understand the significant differences between females and males in this complex disorder.

## 1. Introduction

Eating disorders (EDs) encompass a range of behavioural, potentially life-threatening conditions marked by profound and persistent disruptions in eating habits, along with distressing thoughts and emotions, that affect the quality of life of children and adolescents as well as their families. The disorders include Anorexia Nervosa, avoidant/restrictive food intake disorder, binge eating disorder, bulimia nervosa, other specified feeding and eating disorder, unspecified feeding and eating disorder, pica, and rumination disorder. Diagnostic criteria are defined in the American Psychiatric Association’s Diagnostic and Statistical Manual of Mental Disorders, Fifth Edition, Text Revision (DSM-5-TR), which divides eating disorders into mutually exclusive categories based upon observed symptoms [1,2,3,4,5,6]. The prevalence of eating disorders is reported to be higher in females. According to a retrospective study conducted by Raevuori et al., the prevalence of Anorexia Nervosa in men ranges from 0.16% to 0.3%, with considerable variability in the male-to-female ratio, which ranges from 1:3 to 1:12 [7]. Systematic reviews have estimated the prevalence of Anorexia Nervosa to be 1.4% (0.1–3.6%) in women and 0.2% (0.0–0.3%) in men [8]. However, the incidence of eating disorders in males is rising [9,10], likely at a faster rate than in females [11]. According to data from the Italian Ministry of Health, the incidence of Anorexia Nervosa among women is estimated to be eight new cases per 100,000 people, while it is estimated to be 0.02–1.4 per 100,000 people annually among men [12]. The exact prevalence and incidence of Anorexia Nervosa in pediatric age are difficult to establish as studies often include no uniform sample. Historically, EDs have been considered a female disease as evidenced by the inclusion of amenorrhea as a diagnostic criterion until the publication of DSM-5 [13,14]. Male patients were excluded [11], and this may represent a possible bias and contribute to the underdiagnosis of ED males. Another challenge lies in the difficulty of identifying males affected by Anorexia Nervosa, partially due to the limited acceptance of the EDs by male individuals or the clinicians’ inability to recognize the condition. Furthermore, there is a challenge in incorporating male patients into the diagnostic criteria, as most available questionnaires, which are crucial for diagnosis, are designed for female patients. In contrast to females, males have a more frequent history of obesity or overweight [15], which can progress to an ED due to restrictive diets. This can lead to a delayed diagnosis. Gender medicine is a field of research and practice that focuses on the differences in health outcomes, disease prevalence, and treatment responses between genders. It recognizes that biological, social, and environmental factors influence health and healthcare experiences differently for men and women. Gender medicine promotes personalized healthcare, advocating for research that takes gender differences into account, thereby enhancing the overall quality of care and ensuring that both men and women receive appropriate and effective medical attention tailored to their specific needs [16]. A review of the existing literature suggests that the clinical presentation of males and females is similar in EDs [17,18]. Currently, only a few studies have considered gender differences in eating disorders [19,20,21]. As gender may affect different aspects of health, including diagnosis, treatment, and prevention strategies, it has to be considered in the approach to patients with different diseases, including EDs. Extremely limited data are available in pediatric patients. This retrospective study, conducted on pediatric patients with EDs, aims to evaluate the influence of gender on the clinical, laboratory, and developmental characteristics of EDs in order to gain insight into the influence of gender on the clinical phenotype of EDs in children.

## 2. Materials and Methods

This retrospective study aims to evaluate possible gender differences in a cohort of pediatric patients admitted to the Pediatric Unit of Bambino Gesù Children’s Hospital for EDs between January 2019 and December 2024. For the purpose of this study, we included children and adolescents aged younger than 18 years, admitted with a diagnosis of Anorexia Nervosa to IRCCS Bambino Gesù Children’s Hospital, Rome, Italy, which is a reference third-level centre for pediatric eating disorders in Italy. The study period ranged from 1 January 2019 to 31 December 2024. According to the DSM-5-TR, the diagnosis of Anorexia Nervosa requires each of the following: Restriction of energy intake that leads to a low body weight, given the patient’s age, sex, developmental trajectory, and physical health; intense fear of gaining weight or becoming fat, or persistent behaviour that prevents weight gain, despite being underweight; distorted perception of body weight and shape, undue influence of weight and shape on self-worth, or denial of the medical seriousness of one’s low body weight. Patients over 18 years old or not fulfilling the inclusion criteria were excluded. Informed consent was obtained from all subjects involved in the study. The study was conducted in accordance with the Declaration of Helsinki and approved by the Institutional Ethics Committee (protocol code 3334_OPBG_2024, date of approval 18 September 2024). Diagnosis of EDs was based on DSM-5 [2]. A comprehensive analysis of quantitative and qualitative variables was performed. All subjects included in the study were children and adolescents, from 5 to 18 years of age. An initial cohort of 501 patients (456 females, 91.02%, and 45 males, 8.98%) hospitalized for EDs was evaluated. In the total cohort, the age difference between males and females was evaluated. Due to the higher prevalence of female patients, an age-matching approach was used to address the gender disparity, linking each male patient with three female counterparts of the same age. The final matched cohort consisted of 144 patients (36 males and 108 females, age range 8–17). Clinical and biochemical differences were evaluated in the age-matched population. The quantitative parameters included body mass index (BMI) expressed as standard deviation scores (SDS) at admission and discharge, heart rate, blood pressure, and biochemical parameters. Laboratory values were obtained at the time of hospital admission, coinciding with the diagnosis of EDs, and subsequently during the course of hospitalization. The parameters considered were: blood count (red blood cells, white blood cells, and platelets), electrolytes (sodium, potassium, and calcium), liver function (transaminase and albumin), kidney function (creatinine), ferritin levels, vitamins (B12, folic acid, C, and D), thyroid function (TSH, FT4, and FT3). The evaluation of qualitative parameters was conducted on the following parameters: familiarity with psychiatric disorders, presence or absence of celiac disease, trigger episode (a significant event following which the patient exhibited symptoms of Anorexia Nervosa), comorbidities, generalized anxiety disorder, conduct of elimination, and relapses. In the management of EDs, a multidisciplinary approach, including medical care, nutritional guidance, and psychological support, to help restore healthy eating patterns and address underlying issues, is crucial. The evaluation of differences in treatment was thus also considered. We conducted an analysis of various treatment types, including nutritional rehabilitation and the eventual need for enteral feeding via a nasogastric feeding tube, psychotherapy, and pharmacological therapy.

### Statistical Analyses

All statistical analyses were conducted using the Statistical Software SPSS (IBM SPSS Statistics for Windows, Version 28:0: IBM Corp., Armonk, NY, USA). Given the unequal sample sizes between the two groups (males and females), non-parametric tests were employed to evaluate the data. Specifically, the Mann–Whitney U test was utilized to assess qualitative parameters. Statistically significant differences were defined by *p*-values less than or equal to 0.05. The chi-square test was used to evaluate the association between qualitative parameters. When necessary, the Cramer V index was also reported, with values ranging from 0 to 0.3 indicating low association, 0.3 to 0.6 indicating medium association, 0.6 to 0.9 indicating strong association, and >0.9 indicating perfect association. This approach was adopted to facilitate more meaningful interpretation.

## 3. Results

### 3.1. Age at Presentation Analysis

The age at presentation of EDs was analyzed in a total of 501 patients. The average age of males was found to be 12.65 ± 3.06, while the average age of females was 14.65 ± 2.07. This difference was statistically significant (*p* < 0.001) (Table 1).

### 3.2. Clinical Differences

Clinical features were evaluated among the 144 age-matched patients, and no significant differences were observed, although males seemed to have a worse clinical picture. Indeed, BMI SDS at admission tends to be lower in males compared to females (−2.42 ± 1.92 vs. −2.17 ± 1.77, *p* > 0.05) as well as at the time of discharge (−1.83 ± 1.61 vs. −1.66 ± 1.32, *p* > 0.05). Interestingly, males showed a tendency for better improvement in BMI than females, expressed by a higher delta BMI (0.59 vs. 0.51, *p* > 0.05), and this result was obtained in a shorter time. In fact, females showed a significantly longer hospital stay than males (19.9 vs. 14.8 days, *p* = 0.039). By comparing males to females, no differences in heart rate frequency (63.75 vs. 67.37 bpm, *p* > 0.05) as well as in blood pressure levels, either systolic (PAS) or diastolic (PAD) (102/64 vs. 103/64 mmHg, *p* > 0.05) were found. No significant differences were found in the percentage of abdominal alterations evaluated by means of ultrasound (11.1% in males vs. 12% in females), as well as in the percentage of heart alterations (9.3% in females vs. 11% in males) assessed by cardiac ultrasound (Table 2).

### 3.3. Biochemical Differences

Males and females did not differ in hemoglobin concentration (13.61 vs. 13.59 g/dL, *p* > 0.05), and platelet count (207,110 vs. 209,000/μL, *p* > 0.05), whereas white blood cells were higher in males (5.700/mmc vs. 4810/mmc, *p* < 0.05). No differences in electrolytes were found (between males and females): sodium (139.3 vs. 139.5, *p* > 0.05), potassium (4.48 vs. 4.37, *p* > 0.05), and calcium (9.52 vs. 9.68, *p* > 0.05). Liver function was studied by analyzing alanine transaminase (ALT), aspartate transaminase (AST), and albumin. Males had higher ALT levels (51.25 ± 108.110 vs. 18.86 ± 19.13, *p* = 0.018) as well as AST levels (40.14 ± 63.22 vs. 23.66 ± 9.8 U/L, *p* > 0.05); albumin levels (were not different) (4.7 vs. 4.78, *p* > 0.05) as well as creatinine levels (0.79 vs. 0.74 mg/dL, *p* > 0.05), and iron level (94.27 vs. 87.49 mcg/dL, *p* > 0.05) were not different. Males showed significantly higher ferritin levels (385.87 vs. 196.9 mcg/L, *p* = 0.019) and lower vitamin D levels (23.15 vs. 26.9 ng/mL, *p* = 0.035). No differences in other vitamin levels were found: vitamin B12 (810.31 vs. 733.2 pg/mL, *p* > 0.05), vitamin C (32.82 vs. 32.64 mg/L, *p* > 0.05), and folic acid (6.87 vs. 8.48 ng/mL, *p* > 0.05). Thyroid function did not differ between males and females (Table 3).

### 3.4. Treatment Analysis

We then evaluated a possible different therapeutic approach according to gender. Regarding males, 27.8% did not take any medication; 22.2% took only one; 33.3% took two; 13.9% took three; and only 2.8% took four drugs. Regarding females, 14.8% did not take any drugs; 26.9% took one drug; 38% took two drugs; 12% took three drugs; and 8.3% took four drugs. In particular, the drugs used in the treatment of ED are aripiprazole, sertraline, olanzapine, and fluoxetine. The most commonly used drug in our cohort of patients is aripiprazole alone or sometimes in combination with one of the other above-mentioned (Table 4).

Regarding enteral feeding, 27.8% (n = 10) of males were fed via nasogastric tube (SNG). Of these 10 children, 3 (8.3%) underwent enteral therapy in bolus, while the remaining 7 (19.4%) underwent continuous enteral therapy. No male had any side effects related to NE. 39.8% (n = 43) of females were fed via SNG. (Of these), 21 (19.4%) had enteral therapy in bolus, while the other 23 (21.3%) continued enteral therapy. Only one child had side effects related to enteral nutrition. Cramer’s V-index of SNG therapy was =0.108.

### 3.5. Qualitative Parameters Analysis

Males with familiarity for psychiatric pathologies were 44.4%, while females were 40.7%. Males diagnosed with Celiac Disease were only 1 (2.8%), while females were 4 (3.7%). Males who reported and associated the onset of eating disorder with a specific trigger event were 14 (38.9%), while females were 48 (44.4%). Twelve male patients had comorbidity (33.3%), while 21 female patients (19.4%) presented comorbidity. Generalized anxiety disorder was found in a significantly higher percentage of males than of females (75% vs. 47.2%, *p* = 0.004). Males who reported using elimination behavior (such as self-induced vomiting or purging) were 38.9%, while females were 41.7%. The number of males who had a relapse was 11.1%; in females, relapse tends to occur more frequently (25.9%). Table 5 summarizes qualitative parameters.

## 4. Discussion

EDs are multifactorial diseases that present clinical variability from patient to patient. Early intervention is crucial, as untreated EDs can lead to severe health complications. Gender medicine aims to develop diagnostic and therapeutic strategies that account for sex and gender differences, for a more personalized and tailored medicine. In order to define a gender-specific approach to address the particular needs of each sex, clinical and biochemical features were evaluated in a pediatric cohort of subjects suffering from EDs.

EDs have historically been regarded as conditions predominantly affecting females, but recent studies indicate a growing prevalence among males with a marked increase in the number of male cases.

This study confirms previous data [22] showing a higher prevalence of EDs in females. The previously reported ratio of males to females (M/F) for Anorexia Nervosa ranges from 1:3 to 1:12 [7]. According to our findings, females represent approximately 91% of all cases, whereas males account for about 9% of the total cohort of 501 patients. It remains uncertain whether these data reflect a true incidence or rather a reduced awareness of the manifestation of EDs in the male population.

Interestingly, according to our results, males showed a younger age of the disease onset, and the youngest patient in our whole cohort was a male of approximately 5 years and 3 months. This result is consistent with some previous reports [23,24,25].

Males are often underdiagnosed or misdiagnosed, likely due to stereotypes that consider EDs primarily a female issue. This can lead to delayed treatment and worse outcomes. As EDs can occur early in males, a longer period of misdiagnosis can manifest in this gender and result in a worse clinical phenotype. Consistently, in our cohort, males showed a tendency to lower BMI at the onset of the disease.

Notably, during hospitalization, males showed a higher BMI improvement (+59% vs. +51%) in a shorter time (14.8 vs. 19.9 days), suggesting that boys have an enhanced recovery capacity compared to females. Consistently, the analysis of administered drugs showed that the percentage of males who did not receive any treatment was higher than the percentage of females, suggesting a faster recovery in males and a different therapeutic approach. In addition, only 2.8% of males used up to four drugs at the same time, compared with 8.3% of females. However, it is not clear whether this lower dose of medication is due to faster recovery in males or whether it results from poor clinical experience with male EDs. Currently, there are no available guidelines for the medications that can be used in children and adolescents with EDs, and the experience in males is even more limited.

Given the variety of drugs employed (aripiprazole, sertraline, olanzapine, and fluoxetine) and the limited number of patients receiving pharmacotherapy, no definitive conclusions can be drawn regarding sex-related differences in drug efficacy. This issue warrants investigation in future studies with larger pharmacologically treated cohorts.

Among biochemical parameters, white blood cell counts and transaminase levels were higher in our male subjects. Although higher white blood cell counts were observed in male patients, values remained within the normal range, and the clinical significance of this finding remains unclear. No differences were found in hemoglobin and platelet values. Further research is needed to determine whether subtle hematological variations may have prognostic or diagnostic implications across sexes.

Elevated ALT levels have been associated with a more severe clinical presentation of Anorexia Nervosa, particularly in cases of extremely low BMI [26]. This may be the consequence of hepatic damage. Male patients with lower BMI may present with increased liver involvement, accounting for higher transaminase levels.

Ferritin is usually considered a marker of inflammation [27], and in our cohort, its levels were significantly increased in males. The precise mechanisms underlying ferritin elevation remain to be fully elucidated but may be the consequence of heightened hepatic production of ferritin in response to hepatic stress induced by malnutrition, or the suppression of hepatic activity due to nutritional deficiency [28].

Available data suggest no significant difference in clinical severity of symptoms across the sexes [11]. According to our results, vitamin D levels differ between genders, likely as a consequence of the worse nutritional status displayed by males.

In our cohort, the frequency of the most commonly detected abdominal ultrasound abnormalities (hepatomegaly, gallbladder distension, and biliary tract stones) was comparable between males and females (11.1% vs. 12%). Similarly, no significant sex-based differences were observed in the incidence of cardiac abnormalities, such as pericardial effusion. These results indicate that the instrumental diagnostic evaluation of patients with EDs should be similar across sexes, without the need for sex-specific diagnostic protocols.

Physical and psychiatric comorbidities were more frequent in males than females, in agreement with the data from other studies [29]. For example, autism spectrum disorders have been reported to be more common in males. Among the possible explanations for this difference are a greater vulnerability to prenatal insults or a higher genetic predisposition. In the current study, generalized anxiety disorder was found in 75% of males (vs. 47.2% of females). This is interesting as in the general population, generalized anxiety disorder is more common in females [30], and these findings indicate that males diagnosed with anxiety disorders may warrant closer clinical monitoring due to an increased risk of developing EDs.

The retrospective design of the study, the small sample size, the limited number of male patients, and the lack of long-term follow-up are the main limitations of this study.

Despite the aforementioned limitations, this is, to our knowledge, the first study focusing on gender differences in a population of children and adolescents with EDs. Our data show that there are gender-related differences in the onset, evolution, and management approach of these patients. Further studies are required to understand the underlying mechanisms of these differences, with the aim of developing specific diagnostic and therapeutic algorithms.

## 5. Conclusions

EDs, once considered primarily affecting females, may affect males as well. Gender-sensitive approaches in treatment are essential to address the specific needs of both sexes.

To our knowledge, there are extremely few pediatric data on differences between males and females in the evolution and treatment response of patients with EDs. Our results show a worse clinical course and a younger onset age in male subjects. Males suffering from anxiety disorders should be closely monitored due to the potential development of EDs. Although preliminary, our data suggest the existence of a gender-based difference in therapeutic approaches. Whether this reflects a real clinical difference between males and females or rather a clinician-related bias in treatment selection remains to be determined.

Characterizing the distinct clinical and biochemical features of EDs in males and females may contribute to elucidating the underlying mechanisms of sex-related differences and, concurrently, to identifying critical factors for facilitating early diagnosis.

Overall, our findings underline the need for further investigation performed on larger cohorts to define the influence of gender on clinical decision-making, which may have important implications for a more tailored medicine.

## Figures and Tables

**Table 1:** Age of ED patients according to gender in the whole cohort and in the age-matched cohort.

	Males	Females
Number of the whole cohort Age (yrs ± sds)	45 12.65 ± 3.06	456 14.65 ± 2.07
Age range of the whole cohort yrs	5.3–17 yrs	6.8–18 yrs
Number of the age-matched cohort Age (yrs ± sds)	36 13.6 ± 2.1	108 13.6 ± 2.1
Age range of the age-matched cohort yrs	8–17 yrs	8–17 yrs

yrs: years, sds: standard deviation score.

**Table 2:** Clinical features of patients affected by EDs according to gender.

	Males	Females	*p* Value
BMI SDS at admission	−2.4	−2.2	*p* = 0.5
BMI SDS at discharge	−1.8	−1.7	*p* = 0.9
Heart rate (bpm)	63.7	67.4	*p* = 0.3
PAS (mmHg)	102	103.5	*p* = 0.7
PAD (mmHg)	64	64.4	*p* = 0.6
Duration of recovery (days)	14.8	19.9	*p* = 0.03 *
Abdomen alterations	11.1%	12%	*p* = 0.8
Heart alterations	11.1%	9.3%	*p* = 0.7

BMI: body mass index; SDS: standard deviation score; PAS: systolic blood pressure; PAD: diastolic blood pressure. *p* value < 0.05 was considered statistically significant *.

**Table 3:** Biochemical parameters of ED patients according to gender.

	Normal Range	Males	Females	*p* Value
Hemoglobin (g/dL)	10.5–15.5	13.61	13.59	*p* = 0.9
White blood cells (×10^3^/μL)	4.0–13.50	5.7	4.8	*p* = 0.004 *
Platelets (×10^3^/μL)	150–450	207.11	209.52	*p* = 0.7
Sodium (mEq/L)	136–145	139.91	139.5	*p* = 0.4
Potassium (mEq/L)	3.1–5.1	4.48	4.37	*p* = 0.3
Calcium (mg/dL)	8.8–10.8	9.52	9.68	*p* = 0.08
AST (U/L)	<41	40.14	23.66	*p* = 0.1
ALT (U/L)	<41	51.25	18.86	*p* = 0.018 *
Albumin	3.5–4.2	4.7	4.78	*p* = 0.3
Creatinine (mcg/dL)	Age range	0.79	0.74	*p* = 0.3
Sideremia (mcg/dL)	50–120	94.27	87.49	*p* = 0.5
Ferritin (mcg/L)	13–150	385.87	196.91	*p* = 0.019 *
Vitamin D (ng/mL)	30–100	23.15	26.9	*p* = 0.035 *
TSH (mU/L)	0.51–4.3	2.14	2.20	*p* = 0.5
FT4 (nmoli/L)	0.98–1.64	1.10	1.11	*p* = 0.8

*p* value < 0.05 was considered statistically significant *.

**Table 4:** Therapeutic approach of ED patients according to gender.

Drug Number	Males	Females
0	27.8% n = 10	14.8% n = 16
1	22.2% n = 8	26.9% n = 29
2	33.3% n = 12	38% n = 41
3	13.9% n = 5	12% n = 13
4	2.8% n = 1	8.3% n = 4

**Table 5:** Qualitative parameters of ED patients according to gender.

	Males	Females	*p* Value
Familiarity for psychiatric pathologies	44.4%	40.7%	*p* = 0.6
Celiac Disease	2.8%	3.7%	*p* = 0.7
Trigger episode	38.9%	44.4%	*p* = 0.5
Comorbidities	33.3%	19.4%	*p* = 0.1
Generalized Anxiety Disorder	75%	47.20%	*p* = 0.004 *
Elimination routes	38.9%	41.7%	*p* = 0.7
Relapses	11%	25.90%	*p* = 0.06

*p* value < 0.05 was considered statistically significant *.

## Data Availability

Data supporting reported results are available upon reasonable request by contacting the corresponding authors.

## Abbreviations

EDs	Eating disorders
BMI	Body mass index
SDS	Standard deviation scores
PAS	systolic blood pressure
PAD	diastolic blood pressure
SNG	nasogastric tube
